# DUSP5 suppresses esophageal squamous cell carcinoma by counteracting macrophage-derived AREG-ERK1/2 signaling and disrupting an oncogenic ERK1/2-ELK1-DUSP5 feedback circuitry

**DOI:** 10.1038/s41419-026-08641-0

**Published:** 2026-04-10

**Authors:** Xu Huang, Wenyi Xu, Runze You, Junjie Kuang, Kai Zhu, Jun Li, Jingzhang Li, Yavuz Nuri Ertas, Qiuhong Ma, Maojin Tian, Miao Lin

**Affiliations:** 1 Department of Thoracic Surgery, Zhongshan Hospital, Fudan University, Shanghai, China; 2 Department of Oncology, Liuzhou People’s Hospital, Guangxi Medical University, Liuzhou, Guangxi China; 3 Department of Nuclear, Liuzhou People’s Hospital, Guangxi Medical University, Liuzhou, Guangxi China; 4 Cancer Center, Huizhou First Hospital, Huizhou, Guangdong China; 5 Department of Biomedical Engineering, Erciyes University, Kayseri, 38039 Türkiye; 6 Mini-invasive Interventional Therapy Center, Shanghai East Hospital, Tongji University, Shanghai, China; 7 Department of Clinical Laboratory, Zibo Central Hospital Affiliated to Binzhou Medical University, Zibo, Shandong 255036 China; 8 Zibo Central Hospital Affiliated to Binzhou Medical University, Zibo, Shandong 255036 China

**Keywords:** Cell signalling, Cancer, Lung cancer, Cancer genomics

## Abstract

Dual-specificity phosphatase 5 (DUSP5) is a key regulator of the mitogen-activated protein kinase (MAPK) pathway, with established roles in various types of cancer. However, its function in esophageal squamous cell carcinoma (ESCC) remains unclear. This study combines single-cell transcriptomics with in vitro and in vivo models to investigate the role of DUSP5 in ESCC. Single-cell RNA sequencing revealed tumor-infiltrating myeloid populations, including apolipoprotein C-positive (APOC⁺) macrophages, which interact with tumor cells via the amphiregulin-epidermal growth factor receptor (AREG-EGFR) axis, activating MAPK/extracellular signal-regulated kinase (ERK) signaling to promote tumor growth and immune modulation. We identified a prognostic gene signature linked to these macrophages. DUSP5 expression was downregulated in ESCC tissues, and its overexpression inhibited cell proliferation, induced senescence and apoptosis, and suppressed migration and invasion. In mouse xenografts, overexpression of DUSP5 reduced tumor growth and metastasis. Mechanistically, DUSP5 inhibited ERK1/2 activation, and its tumor-suppressive effects were reversed by ERK1/2 activation. Moreover, ETS Like-1 protein (ELK1), an ERK1/2 downstream transcription factor, was identified as a negative regulator of DUSP5. In a carcinogen-induced model, DUSP5 knockout increased tumor burden, effects reversed by ERK1/2 inhibition. Our findings indicate that the DUSP5-ERK1/2-ELK1 signaling axis, modulated by tumor-infiltrating myeloid cells, contributes to ESCC progression and represents a promising source of biomarkers and therapeutic targets.

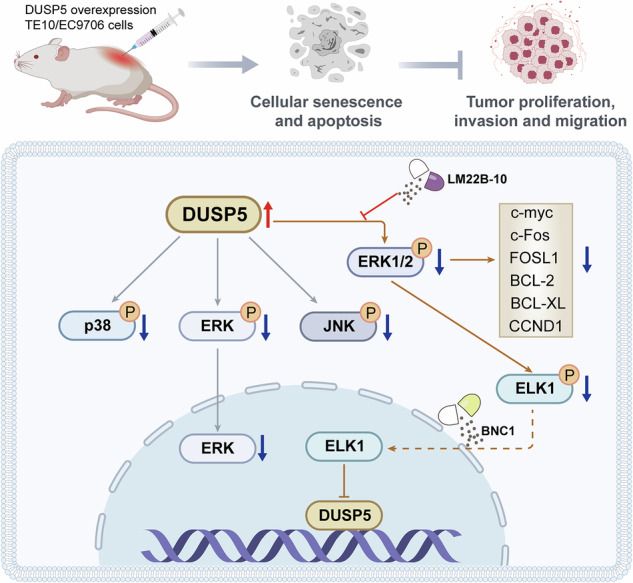

## Introduction

Esophageal squamous cell carcinoma (ESCC) is an aggressive malignancy with unfavorable outcomes, primarily due to early metastasis and resistance to conventional treatments [1, 2]. Despite recent advances in early detection and therapeutic interventions, the molecular mechanisms underlying ESCC progression remain inadequately understood, hindering the development of effective treatment strategies [1, 3, 4]. Recent single-cell transcriptomic studies have unveiled the heterogeneity of myeloid populations within ESCC, identifying distinct macrophage subsets that interact with epithelial tumor cells and influence oncogenic signaling pathways. Evidence suggests that dysregulation of various signaling pathways, particularly the MAPK (Mitogen-Activated Protein Kinase) pathway, is crucial in the initiation and progression of this disease [5, 6]. Among the key regulators of the MAPK pathway, dual specificity phosphatase 5 (DUSP5), extracellular signal-regulated kinase (ERK), and the transcription factor ELK1 have emerged as significant factors influencing the biological behaviors of cancer cells. These molecules are interconnected in a complex regulatory network that impacts tumor growth, metastasis, and resistance to therapy, highlighting their potential as therapeutic targets in ESCC.

DUSP5 is a member of the dual specificity phosphatase family, which regulates the MAPK pathway by dephosphorylating and inactivating MAPK proteins such as ERK [7, 8]. By acting as a negative regulator, DUSP5 helps to maintain cellular homeostasis and prevent unchecked cellular proliferation [9, 10]. Reduced DUSP5 expression is linked to unfavorable outcomes and increased metastatic potential in various cancers, including glioma [11], breast cancer [12], and neuroblastoma [13], implying that it acts as a tumor suppressor. In ESCC, however, the role of DUSP5 remains poorly understood, with few studies investigating its specific impact on the progression and metastasis of the disease. It is hypothesized that DUSP5, by regulating the activation of ERK, might influence the growth, survival, and invasive capabilities of ESCC cells. This makes DUSP5 an interesting candidate for further study in ESCC, particularly as a biomarker or therapeutic target.

The ERK1/2 signaling cascade is a central component of the MAPK pathway [14–16], responsible for transmitting extracellular signals into the cell to regulate essential cellular proliferation, differentiation, and survival [16, 17]. Activation of ERK occurs upon stimulation of receptor tyrosine kinases or G-protein-coupled receptors, leading to the phosphorylation and activation of downstream effectors [18, 19]. Once activated, ERK translocates to the nucleus, where it phosphorylates various transcription factors that drive genes involved in cell cycle progression and survival [20, 21]. In the context of cancer, sustained activation of ERK is often observed, inducing uncontrolled growth and survival of tumor cells. Elevated ERK activity has been associated with increased cell migration, invasion, and resistance to apoptosis, which are hallmarks of aggressive cancers like ESCC [22]. Moreover, ERK activation has been correlated with poor clinical outcomes in ESCC, further emphasizing its role as a key driver of tumorigenesis and metastasis [23–25]. Despite this, the precise mechanisms by which ERK regulates ESCC progression remain unclear, and its therapeutic targeting in this context requires further exploration.

ELK1, a transcription factor activated by ERK, is another pivotal player in the regulation of cancer cell behavior [26]. ELK1 is part of the ETS family and is phosphorylated by ERK at serine 383 in response to various extracellular signals [27]. Once phosphorylated, ELK1 translocates to the nucleus and binds to the promoter regions of target genes, including those involved in cell cycle regulation, apoptosis, and migration. In cancer, ELK1 regulates genes that promote tumor cell proliferation and metastasis [28, 29]. Increased ELK1 activity has been observed in diverse cancers, including breast cancer, glioma, and lung cancer, where it contributes to tumor growth and metastasis [30–33]. In ESCC, it is plausible that the ERK/ELK1 axis may drive genes that facilitate the epithelial-mesenchymal transition (EMT)[34], a process that enhances the migratory and invasive properties of tumor cells. Therefore, the ERK-ELK1 signaling axis represents a crucial node in the network that regulates cancer cell motility, invasion, and metastasis, and its dysregulation could be central to the aggressive nature of ESCC.

The MAPK pathway, particularly the ERK-ELK1 axis, is implicated in diverse cancers, including ESCC, where it influences cell proliferation, survival, and the metastatic spread of tumor cells [35, 36]. ERK activation, through its downstream effects on transcription factors such as ELK1, has the potential to promote both local invasion and distant metastasis, making it a critical player in the progression of ESCC [37]. Despite the extensive research on ERK and ELK1 in other cancer types, their roles in ESCC remain incompletely understood. Our study seeks to bridge this gap by examining the specific role of DUSP5 in the ERK/ELK1 axis and its impact on ESCC progression. This study integrates single-cell transcriptomics and experimental models to dissect the interplay between tumor-infiltrating myeloid cells, the DUSP5-ERK1/2-ELK1 axis, and ESCC progression, aiming to uncover novel therapeutic targets and prognostic biomarkers. By elucidating the molecular interactions between DUSP5, ERK, and ELK1 in the context of ESCC, our research aims to uncover novel therapeutic avenues for managing this challenging and deadly disease.

## Materials and Methods

### Acquisition and processing of transcriptomic data

RNA sequencing data and corresponding clinical information for ESCC patients (*n* = 161) were retrieved from The Cancer Genome Atlas (TCGA) database. These data were used to construct a prognostic model and validate its robustness and accuracy. All RNA-seq data were converted to transcripts per million (TPM) format and log2-transformed before subsequent analyses.

### Acquisition and processing of single-cell RNA-seq data

Single-cell RNA-seq (scRNA-seq) data were obtained from the Gene Expression Omnibus (GEO) under accession number GSE203067, comprising four ESCC tumor samples. Data processing was performed using R software (version 4.1.3), and the Seurat package was employed for downstream analyses. Cells were filtered based on quality control criteria: mitochondrial gene content <20%, hemoglobin gene content < 3%, UMI counts between 200 and 30,000, and gene counts between 200 and 7500. Normalization, selection of the top 2000 highly variable genes, and scaling (regressing out cell cycle effects using vars.to.regress = c(“S.Score”, “G2M.Score”)) were performed using the functions NormalizeData, FindVariableFeatures, and ScaleData in Seurat. Batch effects were corrected using the Harmony algorithm. Dimensionality reduction and clustering were carried out using UMAP and the Louvain algorithm, respectively, both implemented within Seurat. Differentially expressed genes (DEGs) between clusters or cell types were identified using the FindAllMarkers function, with thresholds of adjusted p-value < 0.05, |log2 fold change | > 0.25, and expression in >10% of cells.

### Cell type annotation

Cell type annotation was performed based on canonical markers: Epithelial cells: EPCAM, KRT18, KRT19, CDH; Fibroblasts: DCN, THY1, COL1A1, COL1A2; Endothelial cells: PECAM1, CLDN5, FLT1, RAMP2; T cells: CD3D, CD3E, CD3G, TRAC; NK cells: NKG7, GNLY, NCAM1, KLRD1; B cells: CD79A, IGHM, IGHG3, IGHA2; Plasma cells: JCHAIN; Myeloid cells: LYZ, MARCO, CD68, FCGR3A; Mast cells: KIT, MS4A2, GATA2. UMAP plots, bar charts, and heatmaps were generated to visualize the distribution and expression patterns of annotated cell populations.

### Construction of tumor-related risk signature

Marker genes of C2-type macrophages were selected for univariate Cox regression analysis to identify prognosis-associated genes (*p* < 0.05). A Lasso-Cox regression model was then constructed using the glmnet package, based on the 21 candidate prognostic genes. The predictive performance of the model was evaluated using the timeROC package, and the area under the curve (AUC) was calculated for overall survival at 1-, 3-, and 5-year time points.

### Cell-cell communication analysis

To investigate potential cell-cell communication, the CellChat package was utilized. Normalized gene expression matrices were imported into a CellChat object using the CellChat function. Preprocessing was performed using the default parameters with identifyOverExpressedGenes, identifyOverExpressedInteraction, and projectData. Ligand-receptor interactions were inferred via computeCommunProb, filterCommunication, and computeCommunProbPathway. An aggregated intercellular communication network was generated using aggregateNet.

### Tissue microarray (TMA) and immunohistochemistry (IHC)

A TMA containing data from paired tumor and adjacent non-tumor tissues of 103 ESCC patients was applied for IHC analysis. Representative 1.0-mm cores were extracted from donor blocks using a tissue microarrayer (Alphelys) and arrayed into a recipient block (206 cores total). Sections (4 μm) were cut, deparaffinized in xylene, rehydrated in graded ethanol, and antigen-retrieved in citrate buffer (pH 6.0) by boiling for 10 min. Endogenous peroxidase was blocked with 3% H₂O₂ in methanol (10 min), and non-specific binding with 5% BSA (1 h, 23–25 °C). Slides were incubated overnight at 4°C with primary antibodies against DUSP5 (1:100, Abcam), ERK1/2 (1:200, Cell Signaling Technology), or ELK1 (1:100, Abcam), followed by HRP-conjugated secondary antibodies (1:500, Vector Labs, 1 h, 23–25 °C). Staining was developed with DAB (Vector Labs), counterstained with hematoxylin, and scored independently by two blinded pathologists.

### Cell culture and chemical administration

Human ESCC cell lines KYSE150 (CL-0638), KYSE450 (CL-0990), KYSE30 (CL-0577), and TE-10 (CL-0453) (all provided by Procell Life Science), along with the non-cancerous human esophageal epithelial cell line HET1A (VCH00062, Vicell Biotechnologies) were cultured in RPMI-1640 medium (Gibco) plus 10% fetal bovine serum (FBS; Gibco) and 1% penicillin/streptomycin (Gibco) at 37 °C in a 5% CO₂ incubator. The media were refreshed every 2–3 days. Cells were passaged every 2–3 days and routinely tested for mycoplasma contamination. Cells were treated with the ERK1/2 activator LM22B-10 (HY-104047, MedChemExpress [MCE]) or the ELK1 agonist BNC1 (HY-111000, MCE). The treatment concentrations and durations were selected based on previous studies [38, 39] and validated in our laboratory. Specifically, cells were treated with 10 μM LM22B-10 or 5 μM BNC1 for 24 h, conditions confirmed to induce maximal pathway activation without significant cytotoxicity in TE10 and KYSE30 cell lines.

### Gene intervention in ESCC cells

To overexpress DUSP5 in ESCC cells, a plasmid encoding DUSP5 (GeneChem) was transfected into TE10 and KYSE30 cells using Lipofectamine 2000 (Invitrogen). Stable cell lines were generated by selecting for neomycin resistance with G418 (Geneticin, Invitrogen) at 600 μg/mL for 2 weeks. For knockdown of DUSP5, a lentiviral system expressing DUSP5 short hairpin RNA (shRNA) (Sigma) was used to infect TE10 and KYSE30 cells. All plasmids were confirmed by sequencing before use. Stably infected cells were selected by 1 µg/mL puromycin. Successful gene intervention in cells was confirmed by quantitative polymerase chain reaction (qPCR) or Western blot (WB) analysis prior to subsequent experiments.

### In vivo xenograft model

For subcutaneous tumor formation, 5 × 10^6^ TE10 or KYSE30 cells stably overexpressing DUSP5 were injected into the flank of 6-week-old NOD/SCID mice (Charles River). Tumor volume was gauged weekly using calipers, and tumor weight was determined at the endpoint (35 days). For metastasis assays, cells were injected via the tail vein into NOD/SCID mice, and lungs were harvested at 42 days for metastasis quantification. Lungs were fixed in formalin and stained with hematoxylin and eosin (H&E), and metastatic nodules were counted under a microscope.

### MNNG-induced ESCC mouse model

The MNNG (N-methyl-N’-nitro-N-nitrosoguanidine)-induced ESCC model was established in DUSP5 knockout (KO) mice as previously described with modifications. Briefly, 6-week-old male DUSP5 KO mice (C57BL/6 background) were intragastrically administered with MNNG (1 mg/kg, Sigma-Aldrich) dissolved in saline twice a week for 12 weeks. Mice were monitored for tumor formation throughout the study, with tumor development confirmed by visual inspection and histological analysis at the end of the experiment. After 6 weeks of treatment, the mice were allocated into two groups: one group continued to receive MNNG treatment, and the other group was treated with the ERK1/2-specific inhibitor SCH772984 (Selleck Chemicals) (5 mg/kg, administered orally via gavage twice a week) for the remaining 6 weeks. Tumor burden was assessed by counting visible tumors in the esophagus and by histopathological analysis of tissue sections stained with H&E. Tumor volume and number were recorded, and the tissues were further analyzed for Ki67, C-Cas-3, ERK1/2, and ELK1 expression by IHC.

### Statistical analysis

All statistical analyses and data visualizations were conducted using R software (version 4.1.3). Continuous variables were compared using the Wilcoxon rank-sum test or Student’s *t*-test for pairwise comparisons, as appropriate. For comparisons among multiple groups, one-way ANOVA followed by Tukey’s post-hoc test was used. Categorical variables were compared using the chi-square test. Correlations between continuous variables were assessed using Pearson’s correlation coefficient. Optimal cutoff values for continuous variables were determined using the survminer package. Survival analyses, including univariate and multivariate Cox proportional hazards regression and Kaplan-Meier survival curves, were performed using the survival package.

Data are presented as mean ± standard deviation (SD) or standard error of the mean (SEM), as indicated. A *p*-value < 0.05 was considered statistically significant. All experiments were repeated at least three times, with each group consisting of six animals or biological replicates, unless otherwise specified.

Detailed methodologies for cell proliferation, senescence, apoptosis, migration, and invasion assays, qPCR, WB analysis, immunofluorescence staining, ChIP-qPCR, luciferase reporter, and IHC for markers in mouse tumor tissues are presented in Supplementary methods.

## Results

### Single-cell atlas of ESCC

**Fig. 1:**
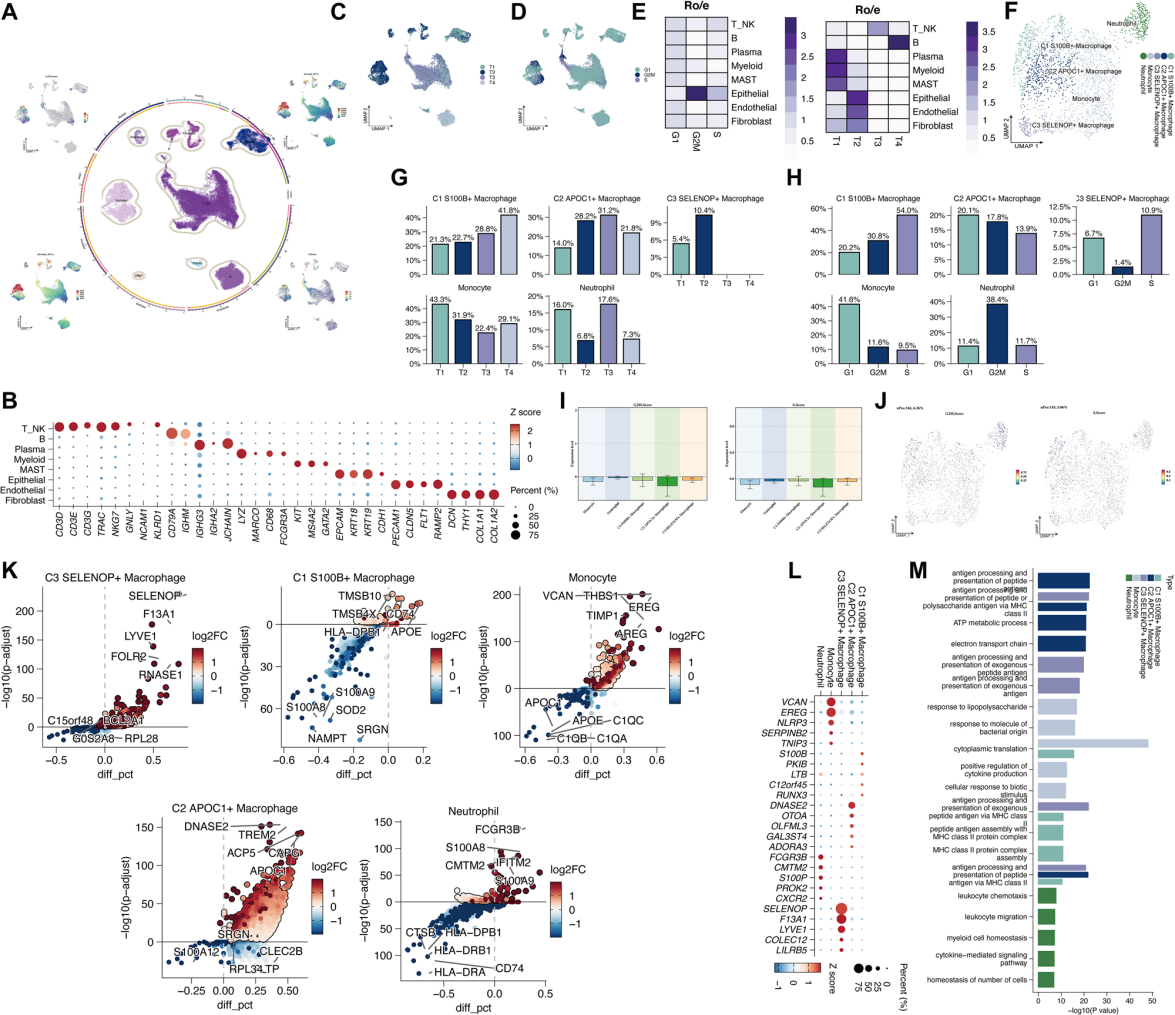
Single-cell transcriptomic atlas of ESCC. **A** UMAP plots showing the distribution of all cells colored by cell type, UMI counts, gene counts, and cell cycle scores (G1, G2/M and S phase). **B** Bubble plot displaying the expression of canonical marker genes across annotated cell types. **C**, **D** UMAP plots illustrating the distribution of cells across different tumor samples (T1-T4) and cell cycle phases (G1, S, G2/M). **E** Heatmaps showing the relative abundance (Ro/e) of each cell type across different samples and cell cycle phases. **F** UMAP plot of re-clustered myeloid cells annotated by subtype. **G**, **H** Bar plots showing the proportion of each myeloid cell subtype across samples and cell cycle phases. **I**, **J** Cell cycle score distribution among myeloid populations shown by bar plots and UMAP visualization. **K** Volcano plot showing differentially expressed genes (DEGs) among myeloid subtypes. **L** Bubble plot showing the expression levels of marker genes specific to each myeloid cell subtype. **M** Bar plots of Gene Ontology (GO) enrichment analysis for top DEGs in each myeloid cell subtype.

Quality control, normalization, dimensionality reduction, and clustering were performed on scRNA-seq data from ESCC tumor samples. Major cell populations were annotated based on canonical markers and included T/NK cells, B cells, plasma cells, myeloid cells, mast cells, epithelial cells, endothelial cells, and fibroblasts. The distributions of UMI counts, gene counts, and cell cycle scores (G2/M and S phases) are shown in the UMAP plot (Fig. 1A). Marker gene expression confirmed the identity of each population (Fig. 1B). The distribution of cell types across the four tumor samples (T1-T4) and cell cycle phases (G1, S, G2/M) is presented in Fig. 1C and 1D. Relative abundance was quantified using the Ro/e method (Fig. 1E). To gain deeper insights into the tumor-infiltrating myeloid compartment, myeloid cells were subsetted and subjected to independent dimensionality reduction and clustering. Three macrophage subpopulations were identified based on specific markers: C1 S100B⁺, C2 APOC⁺, and C3 SELENOP⁺ macrophages (Fig. 1F). Their distribution across tumor samples and cell cycle phases is shown in Fig. 1G and 1H. Interestingly, C1 and C3 subsets exhibited a higher proportion of cells in S phase, suggesting potential proliferative activity. Cell cycle score distributions across myeloid populations are visualized in Fig. 1I and 1J. Marker gene expression profiles for each subtype are presented in Fig. 1L. Additionally, volcano plots of differentially expressed genes (DEGs) among myeloid subtypes, together with Gene Ontology (GO) enrichment analyses, revealed distinct functional programs for each macrophage population (Fig. 1K–M).

### Myeloid cell subpopulation analysis

**Fig. 2:**
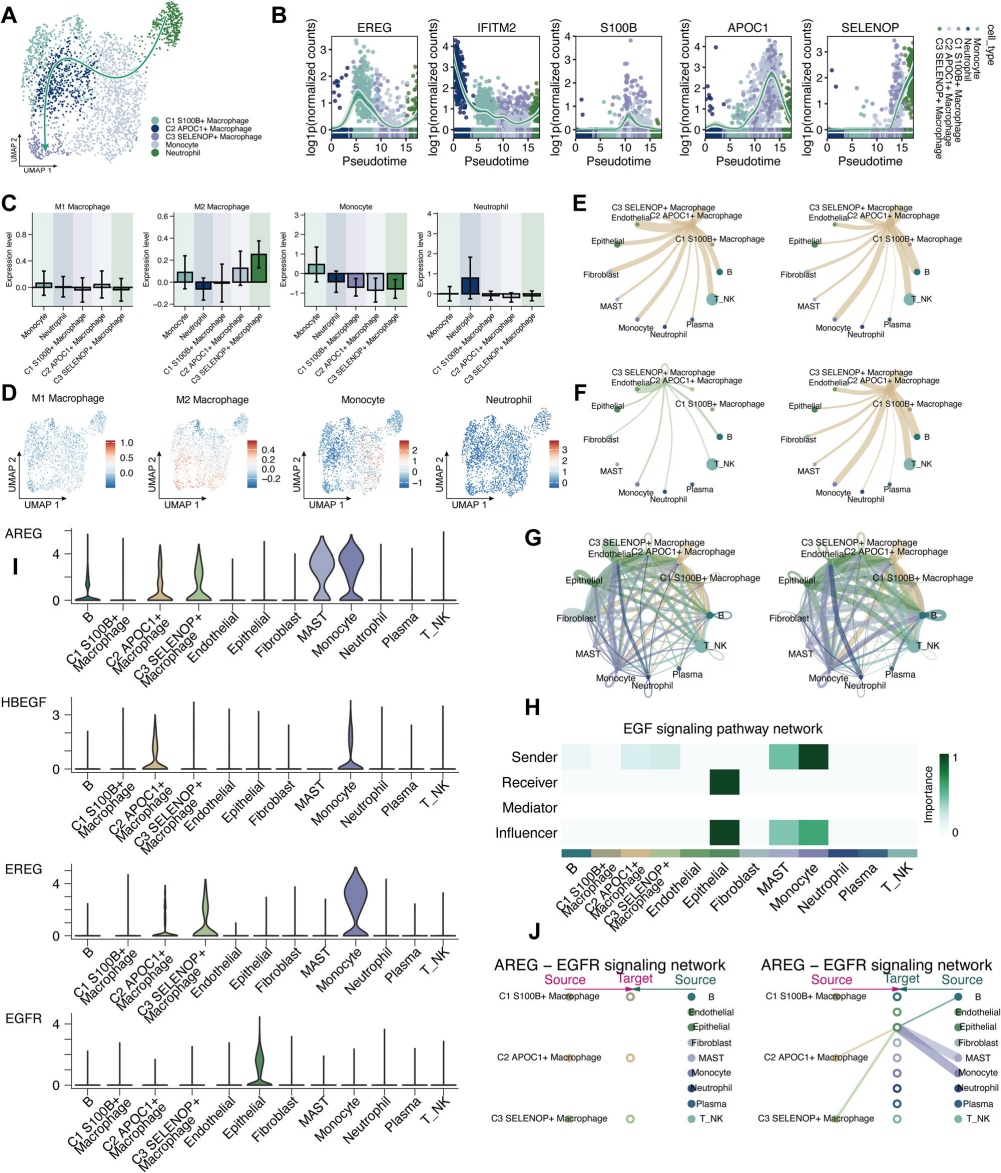
Characterization of myeloid cell subpopulations and intercellular communication. **A** UMAP plot showing pseudotime trajectory inference of myeloid cells using slingshot. Lineage 2 traces a path from neutrophils to monocytes, ending in three macrophage subtypes (C1-C3). **B** Expression of representative marker genes along the inferred trajectory. **C**, **D** Module scores for M1, M2, monocyte, and neutrophil gene signatures shown as bar plots and UMAP projections. **E**–**G** CellChat-inferred intercellular communication networks highlighting interactions between C2/C3 macrophages and epithelial/tumor cells. **H** Heatmap of ligand-receptor pairs within the EGF signaling pathway across cell types. **I** Violin plots showing the expression levels of AREG and EGFR in relevant sender and receiver cell populations. **J** Visualization of AREG-EGFR-mediated intercellular communication network.

Pseudotime trajectory analysis was then performed using the Slingshot package to investigate heterogeneity and differentiation potential of the tumor-infiltrating myeloid compartment. The Lineage 2 revealed a trajectory originating from neutrophils, progressing through monocytes, and terminating in three macrophage subpopulations (C1-C3) (Fig. 2A). Among them, C1 and C2 macrophages clustered closer together in the trajectory, whereas C3 represented a more terminally differentiated state. Expression dynamics of representative marker genes along pseudotime aligned with the spatial distribution of the corresponding cell types (Fig. 2B).

To characterize the functional polarization of macrophages, module scores for M1-like, M2-like monocyte, and neutrophil signatures were calculated using Seurat’s AddModuleScore function. Their distribution, visualized by bar plots and UMAP projections, demonstrated subtype-specific enrichment (Fig. 2C and D). Furthermore, intercellular communication analysis was performed using the CellChat framework to explore potential interactions between myeloid subtypes and other cell populations, revealing strong ligand-receptor interactions between C2 and C3 macrophages and epithelial/tumor cells, particularly via the AREG-EGFR axis (Fig. 2E–G). This axis is known to activate the MAPK/ERK signaling pathway, which is implicated in tumor progression, epithelial remodeling, and cell survival. A heatmap of ligand-receptor pairs within the EGF pathway across all cell types is shown in Fig. 2H. Violin plots confirmed AREG expression in sender cells and EGFR in receiver cells (Fig. 2I). Visualization of AREG-EGFR-mediated cell-cell communication suggests that these macrophage subsets may influence epithelial cell behavior, potentially contributing to immune regulation or stromal remodeling (Fig. 2J).

### Characterization of myeloid cell subtypes

**Fig. 3:**
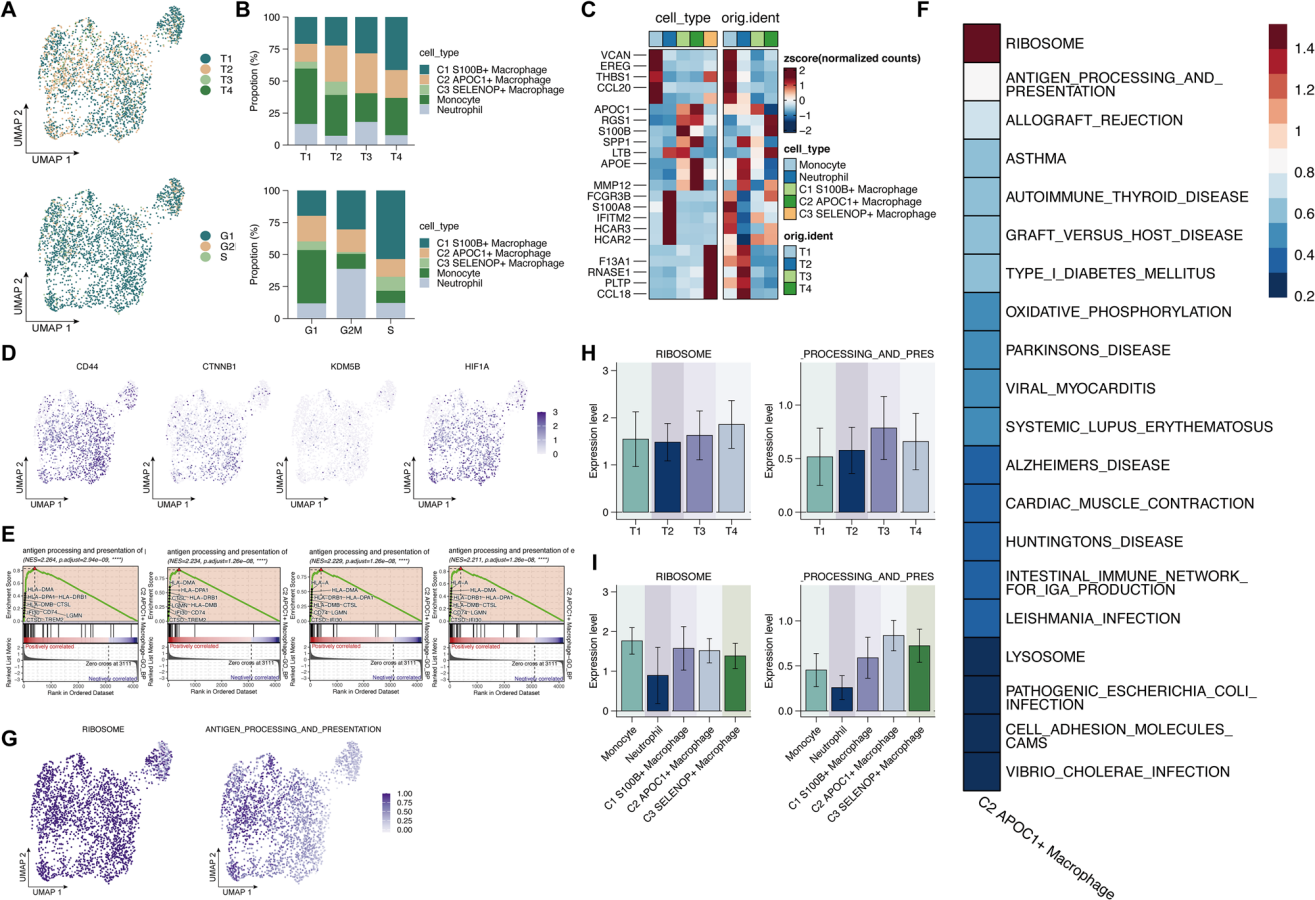
Characterization of myeloid cell subtypes. **A** UMAP plots showing the distribution of myeloid cells by tumor sample and cell cycle phase. B. Bar plots depicting the proportion of each myeloid subtype across samples and cell cycle phases. **C** Heatmap of marker gene expression across myeloid subtypes and tumor samples. **D** UMAP plots showing the expression of four stemness-associated genes (CD44, CTNNB1, KDM5B, HIF1A) in myeloid cells. **E** GSEA plot of GO_BP enrichment results for genes upregulated in the C2 APOC⁺ macrophage subset. **F** Heatmap showing enrichment scores of the top 20 KEGG pathways in C2 macrophages. **G** UMAP plots of enrichment scores for the top two KEGG pathways: ANTIGEN_PROCESSING_AND_PRESENTATION and RIBOSOME. **H**, **I** Bar plots showing enrichment scores of the top two KEGG pathways across tumor samples and myeloid subpopulations.

Building on the previous identification and trajectory analysis of myeloid subpopulations, we further examined their distribution, functional heterogeneity, and transcriptional signatures to gain deeper insights into their potential biological roles in the tumor microenvironment. The distribution of myeloid cells across tumor samples and cell cycle phases was visualized by UMAP, revealing heterogeneous yet structured distribution patterns (Fig. 3A). Bar plots illustrated proportional differences and subtype-specific preferences (Fig. 3B). Next, the expression of representative marker genes for each myeloid subtype, both across subpopulations and within individual samples, was further analyzed. Heatmap analysis of marker gene expression confirmed subtype identity and highlighted sample-specific variations reflecting tumor heterogeneity (Fig. 3C).

Subsequently, expression of stemness-related genes (CD44, CTNNB1, KDM5B, and HIF1A) was determined across the myeloid compartment. UMAP plots revealed variations in these genes across myeloid subtypes, with enrichment in specific macrophage subsets (Fig. 3D), suggesting roles in cell plasticity or differentiation. Notably, Gene Ontology Biological Process (GO_BP) enrichment analysis and GSEA of genes upregulated in C2 APOC⁺ macrophages identified “antigen processing and presentation” as the top-enriched pathway (Fig. 3E). Using the AddModuleScore function, we calculated enrichment scores for KEGG pathways across myeloid cells and identified the top 20 pathways most enriched in the C2 macrophage subset (Fig. 3F). Among these, ANTIGEN_PROCESSING_AND_PRESENTATION and RIBOSOME pathways were the most prominent. UMAP projections illustrated their spatial enrichment, particularly within the C2 cluster (Fig. 3G). The enrichment scores of these two pathways across tumor samples and myeloid subpopulations were further examined. Bar plots revealed that both pathways were highly active in C2 macrophages across samples, further supporting their potential immunomodulatory and protein synthesis-related functions (Fig. 3H and I).

### Comprehensive characterization and clinical relevance of C2 APOC^+^ macrophages and their interaction with epithelial cells

To further complement the findings from the primary pseudotime trajectory analysis (Fig. 2), a deeper exploration of the complete myeloid cell differentiation landscape was conducted. Unlike the simplified lineage presented in Fig. 2, the full myeloid differentiation trajectory revealed multiple branching patterns involving monocytes, neutrophils, and macrophages (Supplementary Fig. 1A), suggesting a more complex differentiation hierarchy. By visualizing lineage-associated marker gene expression across these trajectories, it was observed that C2 APOC⁺ macrophages possess a unique transcriptional signature distinct from classical M1/M2 phenotypes (Supplementary Fig. 1B). Scoring of canonical M1-, M2-, monocyte-, and neutrophil-associated gene modules revealed considerable inter-sample heterogeneity (Supplementary Fig. 1C). CellChat analysis highlighted robust EGF pathway communication between epithelial tumor cells and macrophage subsets, with C2 and C3 macrophages as primary partners (Supplementary Fig. 1D, E). These results position C2 macrophages as key immunomodulatory cells at the tumor-epithelium interface.

Univariate Cox regression of C2-specific DEGs identified 21 genes significantly associated with patient survival (Supplementary Fig. 2A). These were input into a LASSO-Cox regression model, which yielded a 13-gene prognostic signature (Supplementary Fig. 2B, C). The coefficient values of each gene in the model were visualized as a lollipop plot (Supplementary Fig. 2D). Risk stratification of TCGA-ESCC patients revealed distinct clustering of high- and low-risk groups (Supplementary Fig. 2E, F), with strong prognostic performance demonstrated by Kaplan-Meier and time-dependent ROC curves (1-, 3-, and 5-year) (Supplementary Fig. 2G, H). To explore potential biological pathways underlying risk stratification, ssGSEA analysis was performed across hallmark and KEGG pathways from the MsigDB database, uncovering differential immune and metabolic enrichment between risk groups (Supplementary Fig. 2I-K).

To further investigate the impact of C2-epithelial cell interactions, an independent re-clustering of tumor/epithelial cells was further performed (Supplementary Fig. 3A). Based on prior experimental validation, DUSP5 was identified as a tumor suppressor gene of interest, and epithelial cells were stratified into DUSP5-high and DUSP5-low subgroups (Supplementary Fig. 3B). Epithelial subtype distribution varied significantly between the two groups (Supplementary Fig. 3C-D), suggesting potential DUSP5-related transcriptional reprogramming. Expression of canonical epithelial markers confirmed cluster identity (Supplementary Fig. 3E), and AddModuleScore assessment of five ERK/MAPK-related oncogenic pathways revealed differential pathway activity across both epithelial subtypes and DUSP5 expression levels (Supplementary Fig. 3F–I). Volcano plots and GO enrichment analysis of subtype-specific DEGs highlighted functional heterogeneity related to proliferation, immune regulation, and stress responses (Supplementary Fig. 3J, K). Collectively, these analyses highlight the clinical and immunological significance of the C2 APOC⁺ macrophage-epithelial cell axis in ESCC. The unique transcriptional and communication characteristics of C2 macrophages, combined with their prognostic gene signature and interaction with DUSP5-regulated epithelial subtypes, suggest that this macrophage population plays a central role in shaping the tumor microenvironment and may represent a promising therapeutic target.

### DUSP5 overexpression restricts malignant properties of ESCC cells in vitro and in vivo

**Fig. 4:**
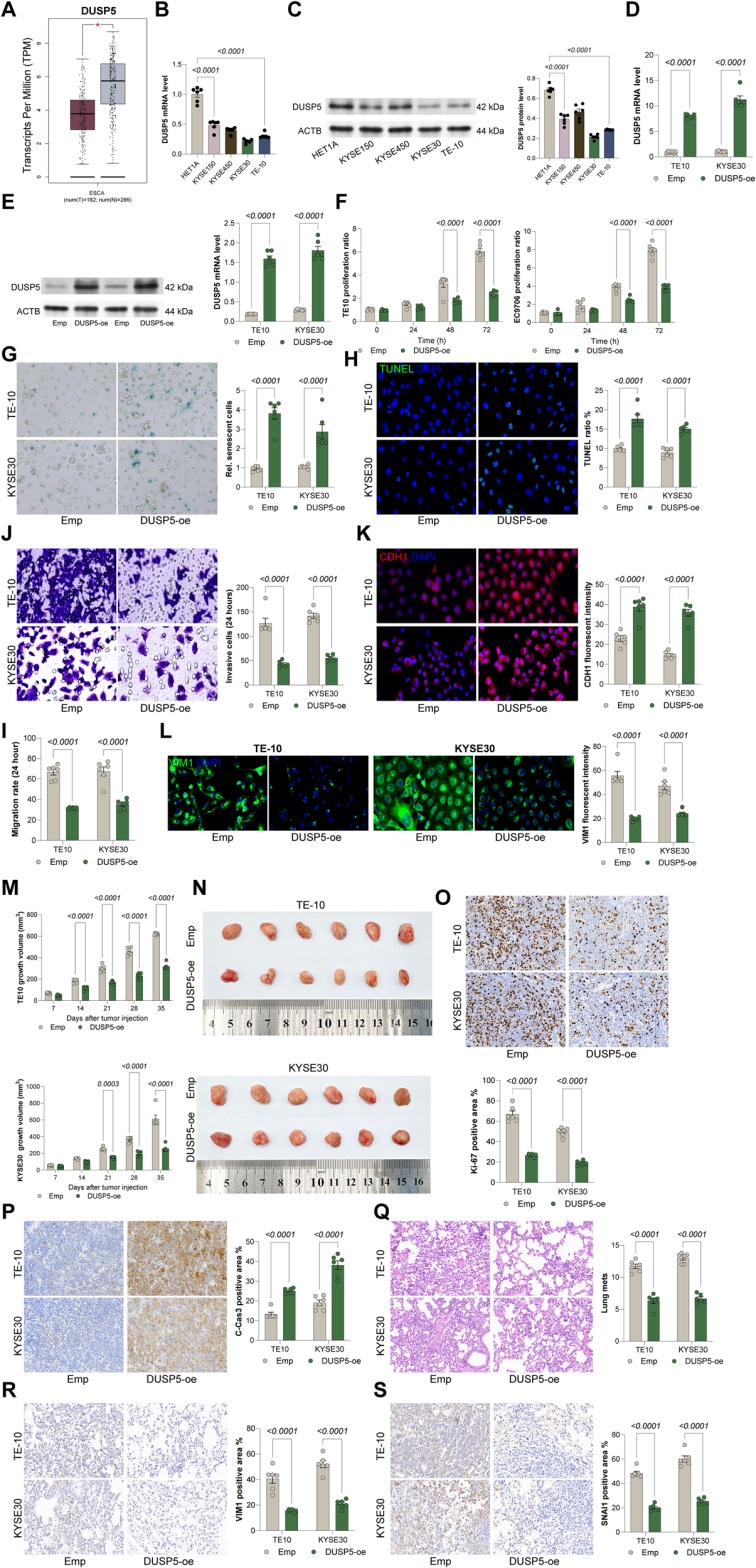
DUSP5 overexpression restricts malignant properties of ESCC cells in vitro and in vivo. **A** Expression of DUSP5 in TCGA-ESCC and GTEx datasets. **B**, **C** qPCR and WB analysis analyses of DUSP5 expression in ESCC cell lines (KYSE150, KYSE450, KYSE30, TE10) and normal esophageal epithelial cells (HET1A). **D**, **E** Confirmation of DUSP5 overexpression in TE10 and KYSE30 cells by qPCR and WB analysis. **F** Cell viability assessed at 0, 24, 48, and 72 h by CellTiter-Glo assay. **G** Senescence detected via β-galactosidase staining in TE10 and KYSE30 cells. **H** Apoptosis measured by TUNEL assay in TE10 and KYSE30 cells. **I** Cell migration evaluated by scratch assay after 24 h. **J** Cell invasion assessed by Transwell assay after 24 h. **K**, **L** Immunofluorescence staining of epithelial marker CDH1 and mesenchymal marker VIM1 in TE10 and KYSE30 cells. **M**, **N** Tumor growth curves and tumor weights from subcutaneous injection of stable DUSP5-overexpressing TE10 or KYSE30 cells in NOD/SCID mice, measured at indicated time points up to day 35. **O**, **P** IHC detection of proliferation marker KI67 and apoptosis marker cleaved caspase-3 (C-Cas3) in xenograft tumors. **Q** Quantification of lung metastatic nodules following tail vein injection of DUSP5-overexpressing ESCC cells in NOD/SCID mice at day 42. **R**, **S** IHC staining of EMT markers VIM1 and SNAI1 in lung metastatic lesions. For animal experiments, each group contained six mice. All cell-based experiments were performed with six independent biological replicates, and three technical replicates were conducted for each biological replicate. Statistical significance was evaluated using one-way (**B**, **C**) or two-way (**D**–**S**) ANOVA with Tukey’s post-hoc test. *P* < 0.05 was considered statistically significant.

Initial analysis of the TCGA-ESCC dataset revealed significantly reduced DUSP5 TPM in tumor tissues compared to normal counterparts (Fig. 4A). A parallel pattern was identified in cell lines, where DUSP5 expression was markedly downregulated in ESCC cell lines (KYSE150, KYSE450, KYSE30, and TE-10) compared to normal HET1A cells (Fig. 4B, C). This evidence suggests a potential tumor suppressor role for DUSP5, which may be lost during in vitro tumor progression. To investigate this, TE10 and KYSE30 cell lines, which exhibited the lowest endogenous DUSP5 expression, were selected to establish stable DUSP5 overexpression models (Fig. 4D, E). Functional assays demonstrated that DUSP5 overexpression significantly inhibited cell proliferation at 24, 48, and 72 h post-transfection (Fig. 4F). Furthermore, DUSP5 induced cellular senescence (Fig. 4G) and increased apoptosis rates (Fig. 4H), consistent with its growth-suppressive effects. Given the critical role of metastasis in cancer progression, the migratory and invasive capacities of these cells were assessed. Transwell assays revealed a significant reduction in cellular motility and invasiveness following DUSP5 overexpression (Fig. 4I, J). Mechanistically, DUSP5 suppressed epithelial-mesenchymal transition (EMT), as evidenced by increased fluorescence intensity of the epithelial marker CDH1 and decreased intensity of the mesenchymal marker VIM1 (Fig. 4K, L).

In vivo validation was performed by subcutaneously injecting TE10 and KYSE30 cells stably overexpressing DUSP5 into immunodeficient NOD/SCID mice. Notably, cells overexpressing DUSP5 exhibited significantly reduced tumorigenic activity compared to control cells, with a noticeable decrease observed from day 14 for TE10 cells and day 21 for KYSE30 cells (Fig. 4M, N). IHC analysis of xenograft tumors further confirmed these results, showing reduced KI67 staining and increased C-Cas3 staining in the DUSP5-overexpressing group (Fig. 4O-P). To assess metastasis potential, ESCC cells with or without DUSP5 overexpression were injected into NOD/SCID mice via tail vein injection. At 42 days post-injection, lungs from mice injected with DUSP5-overexpressing cells exhibited substantially fewer metastatic nodules (Fig. 4Q). Immunofluorescence staining of lung metastatic foci demonstrated marked downregulation of EMT markers VIM1 and SNAI1 in the DUSP5 group (Fig. 4R, S). To further confirm the macrophage-mediated dephosphorylation of ERK via DUSP5, serum starvation was performed on the Vector (Emp) and DUSP5-overexpressing cell lines (TE10 and KYSE30), followed by treatment with recombinant human AREG protein for 0, 0.5, and 1 h. A significant increase in ERK phosphorylation was observed after AREG treatment in the Vector cells. In contrast, AREG had no considerable effect on ERK activation in the DUSP5-overexpressing cells (Supplementary Fig. 4A, B).

Additionally, THP-1 cells were stimulated with PMA and supplemented with IL-4 and M-CSF to induce M2-like macrophage differentiation. Co-culturing with these M2 macrophages significantly enhanced ERK1/2 phosphorylation in ESCC cells. However, DUSP5 overexpression reduced this effect (Supplementary Fig. 4C, D).

### DUSP5 suppresses ESCC via ERK1/2 dephosphorylation, and ERK1/2 activator LM22B-10 abrogates DUSP5-mediated tumor suppression

**Fig. 5:**
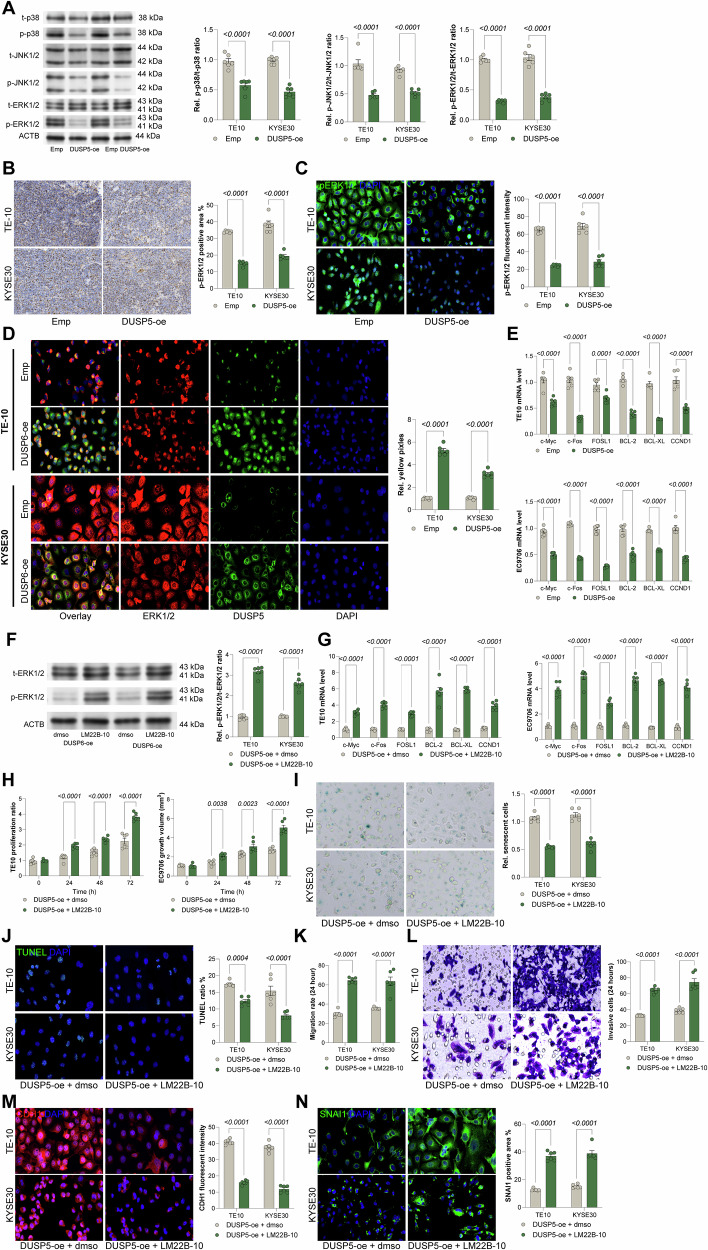
DUSP5 suppresses ESCC via ERK1/2 dephosphorylation, and ERK1/2 activator LM22B-10 abrogates DUSP5-mediated tumor suppression. **A** WB analysis of phosphorylation levels of p38, ERK, and JNK in TE10 and KYSE30 cells with or without DUSP5 overexpression. **B** IHC staining of ERK1/2 phosphorylation in xenograft tumor tissues. C Immunofluorescence analysis showing nuclear localization of ERK1/2 in TE10 and KYSE30 cells. **D** Fluorescence co-localization of DUSP5 and ERK1/2 in TE10 and KYSE30 cells, indicating reduced ERK1/2 activity upon DUSP5 overexpression. **E** qPCR quantification of mRNA levels of ERK1/2 downstream targets: c-Myc, c-Fos, FOSL1, BCL-2, BCL-XL, and CCND1. **F** WB detection of ERK1/2 phosphorylation in DUSP5-overexpressing cells treated with the ERK1/2-specific activator LM22B-10. **G** qPCR analysis of downstream gene expression following LM22B-10 treatment in TE10 and KYSE30 cells. **H** Cell viability assessed by CellTiter-Glo assay at 0, 24, 48, and 72 h in LM22B-10-treated cells. **I** Senescence detected by β-galactosidase staining in LM22B-10-treated TE10 and KYSE30 cells. **J** Apoptosis measured by TUNEL assay in LM22B-10-treated cells. **K** Cell migration evaluated by scratch assay after 24 h of LM22B-10 treatment. **L** Cell invasion assessed by Transwell assay at 24 h following LM22B-10 treatment. **M**, **N** Immunofluorescence staining of epithelial marker CDH1 and mesenchymal marker VIM1 in LM22B-10-treated TE10 and KYSE30 cells. All cell-based experiments were performed with six independent biological replicates, and three technical replicates were conducted for each biological replicate. Statistical significance was evaluated using two-way ANOVA with Tukey’s post-hoc test (**A**–**N**). P < 0.05 was considered statistically significant.

DUSP5 is a well-established negative regulator of the MAPK signaling pathway. In TE10 and KYSE30 cells overexpressing DUSP5, phosphorylation levels of key MAPK family members, including p38, ERK1/2, and JNK, were significantly reduced, with the most pronounced inhibition observed in phosphorylated ERK1/2 (Fig. 5A). Consistent with these in vitro findings, xenograft tumors derived from DUSP5-overexpressing cells also exhibited markedly decreased phosphorylated ERK1/2 levels (Fig. 5B). Since activated ERK1/2 translocates into the nucleus to mediate downstream transcriptional programs, immunofluorescence analyses were conducted, identifying reduced nuclear ERK1/2 localization in DUSP5-overexpressing cells (Fig. 5C). Moreover, fluorescence colocalization assays revealed a significant decrease in yellow pixel intensity, indicative of reduced ERK1/2 activity (Fig. 5D). Consistent with diminished ERK signaling, the expression of canonical ERK1/2 downstream targets, including c-Myc, c-Fos, FOSL1, BCL-2, BCL-XL, and CCND1, was significantly downregulated upon DUSP5 overexpression (Fig. 5E). To determine whether the tumor-suppressive effects of DUSP5 are dependent on ERK1/2 signaling inhibition, DUSP5-overexpressing ESCC cells were treated with the ERK1/2 activator LM22B-10. LM22B-10 treatment effectively restored ERK1/2 phosphorylation and rescued the expression of downstream effector genes (Fig. 5F, G). Functionally, LM22B-10 reversed the anti-proliferative effects of DUSP5 overexpression, decreased cellular senescence, and reduced apoptosis (Fig. 5H–J). Moreover, LM22B-10 treatment enhanced cell migration and invasion capacities compared to vehicle-treated controls (Fig. 5K, L). EMT analysis demonstrated that LM22B-10 promoted the mesenchymal phenotype by decreasing the epithelial marker CDH1 and increasing the mesenchymal marker VIM1 expression (Fig. 5M, N). Together, these data indicate that DUSP5 suppresses ESCC progression primarily through the dephosphorylation and inactivation of ERK1/2, and that pharmacological activation of ERK1/2 by LM22B-10 effectively abrogates DUSP5-mediated tumor-suppressive functions.

### ERK1/2 promotes ELK1 phosphorylation, repressing DUSP5 transcription, and BNC1 negates DUSP5-mediated tumor suppression

**Fig. 6:**
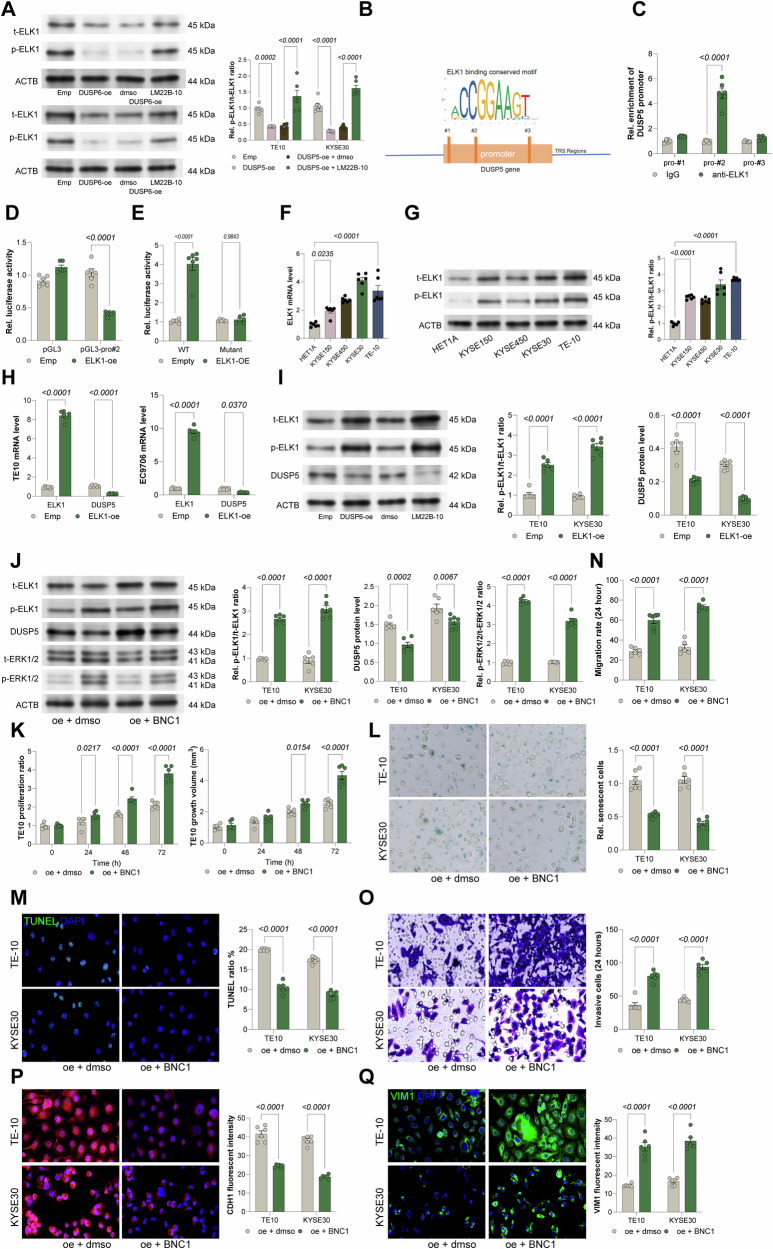
ERK1/2 promotes ELK1 phosphorylation, repressing DUSP5 transcription, and BNC1 negates DUSP5-mediated tumor suppression. **A** WB analysis showing ELK1 phosphorylation levels in the TE10 and KYSE30 cell lines following DUSP5 overexpression or LM22B-10 treatment. **B** JASPAR prediction of ELK1 binding sites in the DUSP5 promoter. **C** ChIP-qPCR for ELK1 enrichment at the DUSP5 promoter in TE10 and KYSE30 cells. **D** Luciferase assay of DUSP5 promoter activity with ELK1 overexpression in HEK293T cells. **E** Luciferase activity of Wild-Type (WT) and Mutant (Mut) DUSP5 promoter constructs in HEK293T cells co-transfected with ELK1. **F**, **G** qPCR and WB of ELK1 mRNA and protein levels in ESCC cell lines (KYSE150, KYSE450, KYSE30, and TE10) and normal esophageal epithelial cells (HET1A). **H**, **I** qPCR and WB detection of DUSP5 and ELK1 in TE10 and KYSE30 cells overexpressing ELK1. **J** WB detection of ERK1/2 phosphorylation in DUSP5-overexpressing cells treated with ELK1 agonist BNC1. **K** Viability of TE10 and KYSE30 cells at 0, 24, 48, and 72 h, in the presence of BNC1 treatment, measured by Celltiter Glo assays. **L** Senescence by β-galactosidase staining in cells treated with BNC1. **M** Apoptosis measured by TUNEL assay after BNC1 treatment. **N** Scratch assay assessing migration at 24 h post-BNC1 treatment. **O** Transwell invasion assay at 24 h post-BNC1 treatment. **P**, **Q** Immunofluorescence for epithelial marker CDH1 and mesenchymal marker VIM1 in BNC1-treated cells. All cell-based experiments were performed with six independent biological replicates, and three technical replicates were conducted for each biological replicate. Statistical significance was evaluated using one-way (**F**, **G**) or two-way (**A**, **C**–**E**, **H**–**Q**) ANOVA with Tukey’s post-hoc test. *P* < 0.05 was considered statistically significant.

As a critical downstream effector of ERK1/2 signaling, the transcription factor ELK1 emerged as a potential regulator within this feedback loop. In DUSP5-overexpressing TE10 and KYSE30 cells, phosphorylation of ELK1 was significantly reduced, consistent with suppressed ERK1/2 activity. Notably, treatment with the ERK1/2 activator LM22B-10 restored ELK1 phosphorylation levels (Fig. 6A), confirming ELK1 as a key downstream effector of ERK1/2 in this context. Bioinformatic analysis using the JASPAR database predicted multiple ELK1 binding sites within the DUSP5 promoter region (Fig. 6B). ChIP-qPCR revealed that ELK1 binding was most enriched at the second predicted binding site (Fig. 6C). Complementary luciferase reporter assays confirmed that ELK1 overexpression significantly repressed DUSP5 promoter activity, supporting a negative feedback mechanism (Fig. 6D). Furthermore, a mutant DUSP5 promoter luciferase reporter (DUSP5-Mut), in which the core ELK1 binding sequence (Site #2 identified in Fig. 6C), was constructed. Dual-luciferase reporter assays comparing the wild-type and mutant promoters in HEK293T cells co-transfected with ELK1 showed no significant change in luciferase activity in the mutant cells (Fig. 6E). Consistent with its oncogenic role, ELK1 mRNA and protein levels, as well as its phosphorylation status, were elevated in ESCC cell lines compared to controls (Fig. 6F, G). Further functional studies demonstrated that ELK1 overexpression in TE10 and KYSE30 cells resulted in a marked reduction in DUSP5 mRNA and protein expression (Fig. 6H, I), confirming the transcriptional repression of DUSP5 by ELK1.

To validate the functional relevance of this regulatory axis, DUSP5-overexpressing cells were treated with BNC1, a specific ELK1 agonist. BNC1 treatment increased ELK1 phosphorylation, decreased DUSP5 expression, and restored ERK1/2 phosphorylation (Fig. 6J). Functionally, BNC1 significantly enhanced cell proliferation, reduced cellular senescence, and attenuated apoptosis in DUSP5-overexpressing cells (Fig. 6K–M). Moreover, BNC1 treatment promoted cell migration and invasion (Fig. 6N, O) and facilitated EMT, as indicated by altered expression of canonical markers (Fig. 6P, Q). Collectively, these findings establish an ERK1/2-ELK1-DUSP5 negative feedback loop, in which ERK1/2-mediated phosphorylation of ELK1 represses DUSP5 transcription, thereby modulating tumor progression. Activation of ELK1 by BNC1 disrupts DUSP5’s tumor-suppressive effects, highlighting the pivotal role of this axis in ESCC pathogenesis.

### The DUSP5-ERK1/2-ELK1 axis affects ESCC progression and correlates with prognosis in ESCC patients

**Fig. 7:**
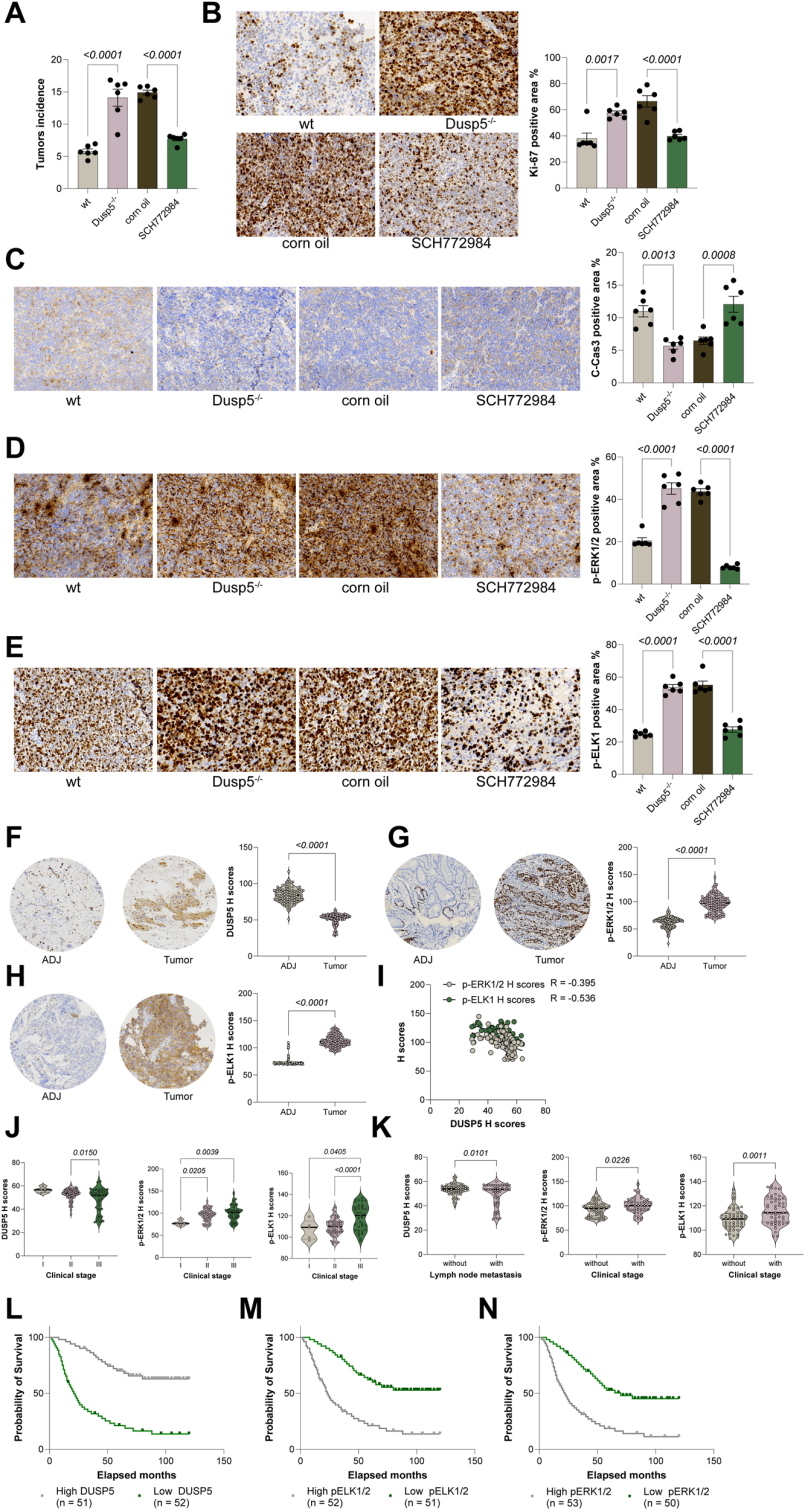
DUSP5-ERK1/2-ELK1 axis drives ESCC progression and associates with clinical outcomes. **A** Tumor counts in esophagus of Dusp5 knockout (KO) mice treated with MNNG and ERK1/2 inhibitor SCH772984. **B**, **C** IHC staining for proliferation marker Ki67 and apoptosis marker C-Cas3 in tumor tissues from mouse groups. **D**, **E** IHC staining of ERK1/2 and ELK1 phosphorylation in tumors from different mouse groups. **F**–**H** IHC analysis of DUSP5, ERK1/2, and ELK1 expression in tumor vs adjacent normal tissues from 103 ESCC patients. **I** Pearson correlation of DUSP5 staining intensity with ERK1/2 and ELK1 levels in patient samples. **J**, **K** Comparison of DUSP5, ERK1/2, and ELK1 expression across ESCC patient groups stratified by tumor stage and lymph node metastasis. **L**–**N** Correlations between DUSP5, ERK1/2, and ELK1 and overall survival in ESCC patients analyzed using Kaplan-Meier analysis. For animal experiments, each group contained six mice (*n* = 6). TMA included data from 103 patients with ESCC. Statistical significance was evaluated using one-way ANOVA with Tukey’s post-hoc test (**A**–**E**, **J**, **K**), or compared by Student’s *t* tests. *P* < 0.05 was considered statistically significant.

To further validate the functional role of the DUSP5-ERK1/2-ELK1 signaling axis in vivo, a mouse model with primary ESCC was established in either WT or Dusp5 knockout (KO) mice via MNNG administration. Compared with WT mice, Dusp5 KO mice developed significantly higher tumor multiplicity, accompanied by elevated Ki67 staining and decreased cleaved Caspase-3 staining in tumor tissues (Fig. 7A–C). Consistent with the proposed mechanism, phosphorylation levels of ERK1/2 and ELK1 were markedly increased in tumors from Dusp5 KO mice (Fig. 7D, E). Notably, pharmacological inhibition of ERK1/2 using SCH772984 in Dusp5 KO mice significantly reduced tumor burden, suppressed cellular proliferation, and enhanced apoptosis (Fig. 7A–C), underscoring the critical involvement of ERK1/2 in mediating the oncogenic effects of DUSP5 loss. To explore the clinical relevance of this signaling axis, a TMA comprising tumor and adjacent non-tumor samples from 103 ESCC patients was analyzed. IHC assays demonstrated that DUSP5 expression was significantly downregulated in tumor tissues, whereas both ERK1/2 and ELK1 exhibited markedly higher expression levels in tumors compared to adjacent normal tissues (Fig. 7F–H). Notably, a strong inverse correlation was observed between DUSP5 staining intensity and the levels of ERK1/2 and ELK1 (Fig. 7I), suggesting that DUSP5 loss is associated with increased activation of the ERK1/2-ELK1 pathway in ESCC. Further stratification based on clinical parameters revealed that patients with advanced tumor stages or lymph node metastasis exhibited significantly lower DUSP5 expression alongside elevated ERK1/2 and ELK1 levels (Fig. 7J, K). Furthermore, we observed a significant positive correlation between low DUSP5 expression and overall survival in ESCC, while ERK1/2 and ELK1 expression were significantly negatively correlated with OS in these patients (Fig. 7L–N). These data indicate that the DUSP5-ERK1/2-ELK1 axis is closely linked to ESCC progression and metastasis and may serve as a valuable prognostic biomarker for disease severity.

## Discussion

This study examines the crucial role of DUSP5 in controlling the ERK1/2-ELK1 axis in ESCC, highlighting its influence on tumor progression, metastasis, and treatment resistance. Our findings reveal a significant downregulation of DUSP5 in both ESCC cell lines and patient tissues. Overexpression of DUSP5 remarkably reduced ESCC cell proliferation, migration, and invasion, and increased apoptosis and cellular senescence. These observations point to DUSP5 as a potential tumor suppressor in ESCC, where its loss leads to sustained ERK1/2 activation and an aggressive cancer phenotype. Notably, this study links DUSP5 inactivation to the enhanced activation of the ERK1/2 pathway, which drives ESCC progression through key downstream effectors, including ELK1, a major transcription factor involved in cell migration and invasion.

Our results are consistent with previous studies in other malignancies, which have shown that DUSP5 negatively regulates ERK1/2 signaling. For instance, in lung adenocarcinoma, Wang et al. reported a notable reduction in DUSP5 expression, and its loss was correlated with persistent ERK1/2 activation, promoting enhanced migratory and invasive behaviors in cancer cells [40]. Similarly, studies in colorectal cancer and breast cancer have illustrated that DUSP5 downregulation contributes to heightened ERK1/2 activation, which is linked with tumor progression and unfavorable outcomes [41**–**43]. These findings align with our own observations in ESCC, where the overexpression of DUSP5 resulted in reduced ERK1/2 phosphorylation and limited cell migration and invasion. Additionally, the downregulation of key pro-survival and pro-proliferative genes c-Myc, BCL-2, and CCND1, which ERK1/2 regulates, further supports DUSP5 as a critical suppressor of the MAPK pathway in ESCC. These findings also emphasize the universality of DUSP5’s tumor-suppressive role across different cancers.

In the context of the MAPK pathway, ERK1/2 is well-known for its involvement in regulating cell proliferation, survival, and migration [35, 36]. Our data demonstrate that DUSP5 overexpression suppresses ERK1/2 phosphorylation and its nuclear translocation, ultimately inhibiting ELK1 activation. As a transcription factor activated by ERK1/2, ELK1 regulates the expression of genes that drive tumor progression, including those involved in cell cycle progression and survival [30, 32, 35]. In our study, DUSP5 knockdown triggered enhanced ERK1/2 activation and subsequent phosphorylation of ELK1, thereby promoting the migratory and invasive abilities of ESCC cells. This finding aligns with the results of other studies, where sustained ERK1/2 signaling induced EMT and metastatic potential in various cancers [44, 45]. For example, several studies demonstrated that persistent ERK1/2 activation in lung cancer cells led to EMT, facilitating migration and invasion [46**–**48]. Similarly, the ERK1/2-ELK1 axis has been implicated in regulating genes that promote survival and proliferation, such as BCL-2 and CCND1, in pancreatic cancer [49], findings that align with the molecular changes observed in our ESCC model.

ELK1 is a critical transcription factor downstream of ERK1/2 that facilitates genes implicated in cell cycle regulation, apoptosis, and migration. In our study, overexpression of ELK1 in ESCC cells reversed the tumor-suppressive effects of DUSP5 overexpression, leading to enhanced tumor growth and metastasis. This observation underscores the pivotal role of the ERK1/2-ELK1 signaling axis in driving ESCC malignancy. Previous articles have also highlighted the involvement of ELK1 in other cancers, including breast and colorectal cancers, where its activation fosters metastasis through EMT-related genes [30**–**32, 35]. For instance, in breast cancer, the ERK1/2-ELK1 axis regulates BCL-2 and CCND1, contributing to enhanced survival and proliferation of cancer cells [50]. Similarly, in colorectal cancer, ELK1 is essential for the transcriptional activation of genes that drive tumor metastasis 31289617. These studies reinforce the importance of the ERK1/2-ELK1 pathway in various malignancies and corroborate the significance of our findings in ESCC.

Moreover, our research offers valuable insights into the potential therapeutic implications of modulating the DUSP5-ERK1/2-ELK1 axis in ESCC. We found that the activation of ERK1/2 or ELK1 could counteract the tumor-suppressive effects of DUSP5 overexpression, resulting in enhanced tumor growth and metastasis. These findings suggest that targeting the DUSP5-ERK1/2-ELK1 pathway may be a promising strategy for ESCC treatment, particularly in advanced stages when tumors are highly metastatic and resistant to conventional therapies. Previous studies have also explored the therapeutic potential of the MAPK pathway in cancer [51]. For example, the ERK1/2 inhibitor SCH772984 has demonstrated preclinical efficacy in suppressing tumor growth in lung and colorectal cancer models [52]. Our results suggest that combining MAPK inhibitors with strategies to restore DUSP5 function could offer a novel approach to treating ESCC and other cancers with dysregulated MAPK signaling.

Furthermore, the potential for combining DUSP5 restoration with MAPK inhibitors is particularly promising given the limited effective therapies available for advanced-stage ESCC [53, 54]. Recent studies have suggested that MAPK inhibitors, particularly those targeting ERK1/2, hold promise in reversing resistance to conventional treatments such as chemotherapy and radiation [55, 56]. Our findings that DUSP5 overexpression sensitizes ESCC cells to these therapies by modulating the ERK1/2-ELK1 axis further support the exploration of combination therapies for ESCC. Notably, the activation of the ERK1/2 pathway contributes to resistance to chemotherapy and targeted therapies in a variety of cancers [57, 58], highlighting the therapeutic importance of restoring DUSP5 expression or inhibiting the ERK1/2-ELK1 axis to overcome this resistance. In addition to the direct tumor cell-intrinsic effects of DUSP5 via the ERK1/2-ELK1 axis, our integrated bioinformatics analyses uncovered a potential role of DUSP5 in modulating the tumor immune microenvironment. Single-cell RNA sequencing of ESCC tissues revealed an enrichment of APOC⁺ tumor-associated macrophages, which are known to influence tumor progression and immune suppression. Our data suggest that DUSP5 expression correlates inversely with pro-tumorigenic macrophage signatures, implying that DUSP5 might also exert anti-tumor effects through remodeling the tumor microenvironment. These findings extend the functional scope of DUSP5 beyond cancer cell-autonomous pathways, offering insights into how DUSP5 restoration could synergize with immunomodulatory strategies in ESCC treatment. However, due to time constraints, this study did not further investigate the precise mechanisms contributing to the loss of DUSP5 in ESCC. Previous studies have indicated that promoter CpG island hypermethylation-mediated epigenetic silencing may play a significant role. For instance, in gastric cancer, DUSP5 promoter hypermethylation occurs in approximately 40-50% of tumors and correlates with advanced disease stages and poor prognosis, suggesting it as a driver of tumorigenesis independent of transcriptional repressors like ELK1 [59]. Similar epigenetic downregulation has been observed in colorectal cancer, where DUSP5 promoter methylation is associated with CpG island methylator phenotype (CIMP-high) tumors, further linking it to aberrant MAPK signaling [42]. Further studies are needed to verify whether this mechanism contributes to DUSP5 loss in the context of ESCC.

In conclusion, while the tumor-suppressive roles of DUSP5 and the oncogenic roles of the ERK1/2-ELK1 cascade have been reported in several malignancies, the present study establishes several layers of novelty specific to ESCC. First, we uncover an oncogenic positive feedback loop in which phosphorylated ELK1, traditionally viewed only as a downstream effector of ERK1/2, also functions as a direct transcriptional repressor of DUSP5. This creates a self-reinforcing circuit: loss of DUSP5 leads to sustained ERK1/2 activation, increased ELK1 phosphorylation and nuclear translocation, and further repression of DUSP5 transcription, thereby locking tumor cells into a state of hyperactive MAPK signaling and aggressive behavior. Second, by integrating single-cell RNA sequencing of primary ESCC specimens, we identify an extrinsic upstream trigger of this intrinsic feedback loop: a distinct APOC1⁺ tumor-associated macrophage subpopulation that abundantly secretes AREG. AREG-mediated EGFR activation in tumor cells sustains ERK1/2 hyperactivity, which DUSP5 usually counteracts through dephosphorylation. These findings underscore the potential for targeting the DUSP5-ERK1/2-ELK1 axis as a therapeutic strategy for ESCC, providing a basis for future clinical trials investigating MAPK inhibitors and DUSP5 restoration in the treatment of this aggressive cancer.

## Supplementary information

Supplementary Material

Western Blot

qPCR.rar
