# Supplementary material for: DUSP5 suppresses esophageal squamous cell carcinoma by counteracting macrophage-derived AREG-ERK1/2 signaling and disrupting an oncogenic ERK1/2-ELK1-DUSP5 feedback circuitry
Source: Cell Death Dis. 2026 Apr 10;17(1):498. doi: 10.1038/s41419-026-08641-0 (PMC13187339; doi:10.1038/s41419-026-08641-0)
Supplement: Supplementary file 2 — Western Blot [file 41419_2026_8641_MOESM2_ESM.pdf]

1C

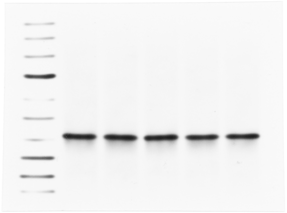

actin

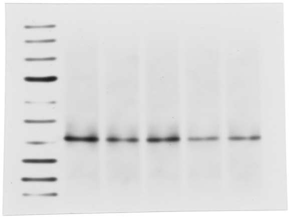

DUSP5

1E

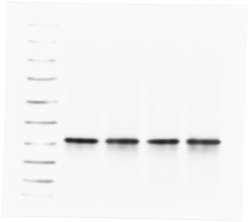

actin

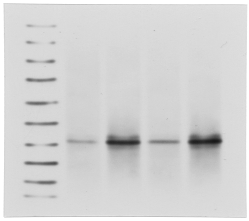

DUSP5

3A

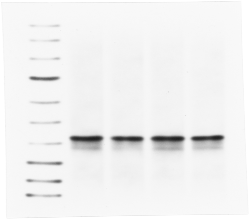

actin (1)

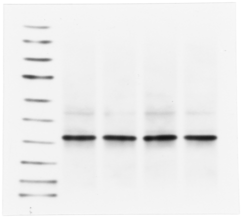

actin (2)

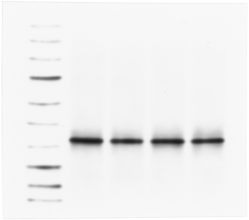

actin (3)

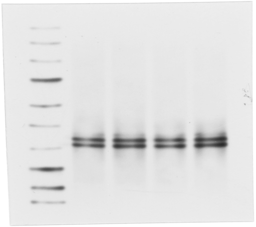

ERK

3A

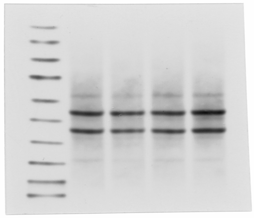

JNK

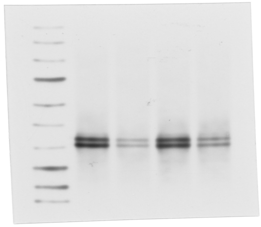

p−ERK

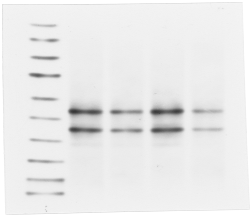

p−JNK

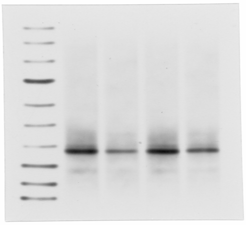

p−p38

3A

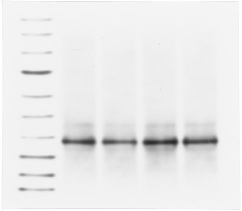

p38

4A

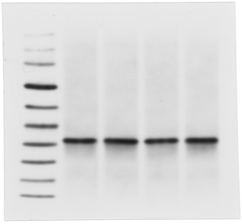

actin

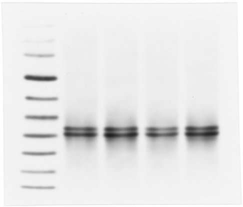

ERK

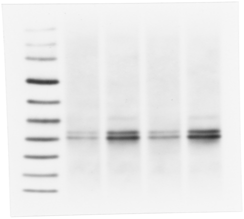

p−ERK

5A1

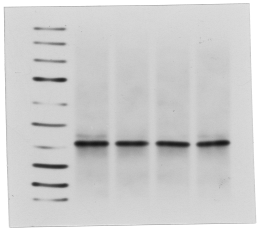

actin

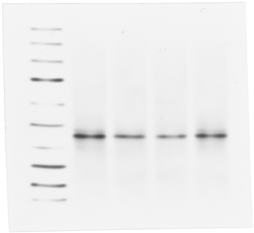

ELK1

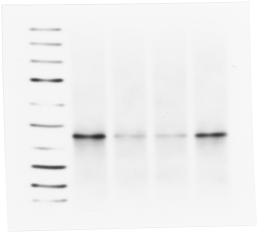

p−ELK1

5A2

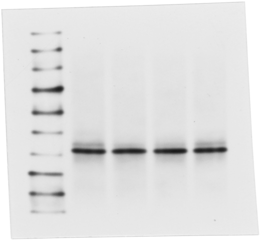

actin

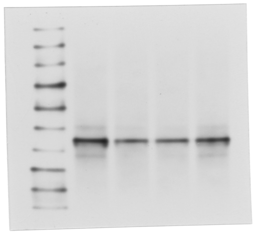

ELK1

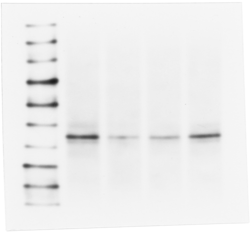

p−ELK1

5F

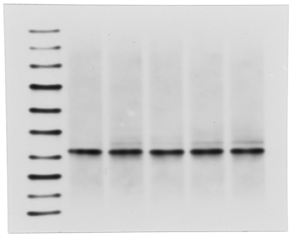

actin

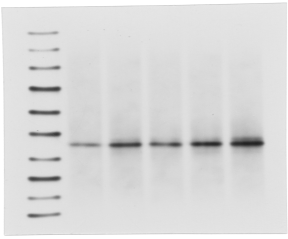

ELK1

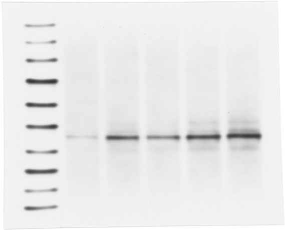

p−ELK1

5H

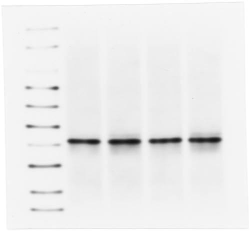

actin

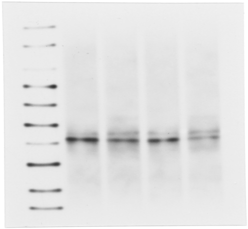

DUSP5

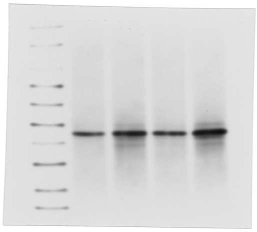

ELK1

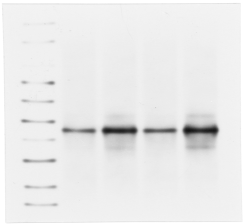

p−ELK1

6A

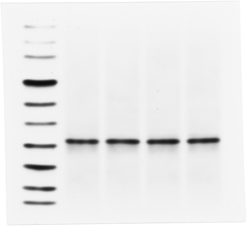

actin (1)

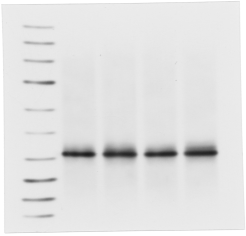

actin (2)

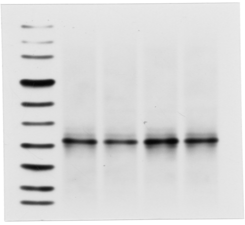

DUSP5

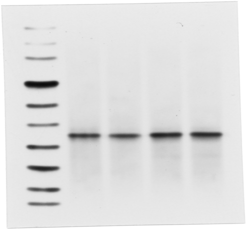

ELK1

6A

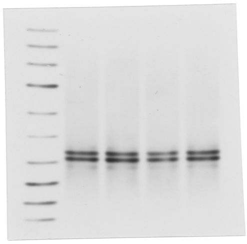

ERK

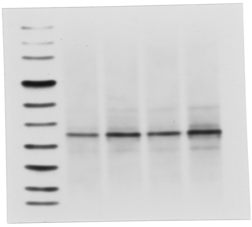

p−ELK1

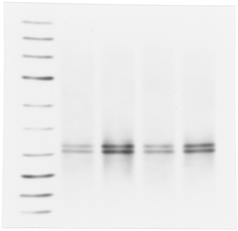

p−ERK
