# Supplementary material for: DUSP5 suppresses esophageal squamous cell carcinoma by counteracting macrophage-derived AREG-ERK1/2 signaling and disrupting an oncogenic ERK1/2-ELK1-DUSP5 feedback circuitry
Source: Cell Death Dis. 2026 Apr 10;17(1):498. doi: 10.1038/s41419-026-08641-0 (PMC13187339; doi:10.1038/s41419-026-08641-0)
Supplement: Supplementary file 1 — Supplementary Material [file 41419_2026_8641_MOESM1_ESM.docx]

**Supplementary materials and methods**

**Cell proliferation assays**

Cell proliferation was assessed using the CellTiter-Glo assay (Promega). Briefly, cells were cultured in 96-well plates (3,000 cells per well) for 24, 48, 72, and 96 h. At each time point, 100 μL of Celltiter-Glo reagent was added to each well, and the luminescence was measured with a microplate reader (BioTek Instruments). Proliferation was normalized to the background level of each cell line.

**Cell senescence assays**

Senescence was examined with the Senescence β-Galactosidase Staining Kit (Cell Signaling Technology, #9860). TE10 and KYSE30 cells were fixed and cultured in a staining solution containing X-gal at 37℃for 12 h. Senescent cells were identified by blue staining. The percentage of senescent cells was gauged by counting at least 500 cells per replicate in three independent experiments.

**Apoptosis assays**

Apoptosis was examined by Terminal deoxynucleotidyl transferase (TdT)-mediated dUTP nick end labeling (TUNEL) using the DeadEnd Fluorometric TUNEL System (Promega). TE10 and KYSE30 cells were fixed in 4% paraformaldehyde and added with TUNEL reaction mixture at 37℃for 60 min. After washing, cells were stained with DAPI for nuclear visualization. Apoptotic bodies were quantified by counting TUNEL-positive cells. At least 200 cells were counted per condition, and results were delineated as the percentage of apoptotic cells.

**Transwell assays**

For cell migration, a scratch assay was conducted. TE10 and KYSE30 cells were cultured in 6-well plates and allowed to form a confluent monolayer. A scratch was made with a sterile 200-μL pipette tip, and cells were cultured in serum-free medium. Migration was recorded at 0 and 24 h under a light microscope (Olympus). The percentage of wound closure was gauged by comparing the initial and final gap areas.

For cell invasion, a Transwell assay was conducted. Cells were resuspended in serum-free medium and seeded in the upper chamber of a Transwell insert (8-μm pore size; Corning) coated with Matrigel (BD Biosciences). The lower chamber contained RPMI-1640 medium with 10% FBS. After culture for 24 h, non-invading cells were removed, and the invading cells were fixed in 4% paraformaldehyde, stained with crystal violet, and counted under a microscope.

**WB analysis**

Proteins were extracted from cells using RIPA buffer (Thermo Fisher) with protease and phosphatase inhibitors (Sigma). The protein concentration was quantitated utilizing the BCA Kit (Thermo Fisher). Equal amounts of protein (30 μg) were separated by SDS-PAGE and moved onto PVDF membranes (Millipore). After blockade with 5% non-fat milk, membranes were probed with primary antibodies (DUSP5, ERK1/2, phospho-ERK1/2, p38, phospho-p38, JNK, phospho-JNK, Ki67, C-Cas-3, ELK1, and phospho-ELK1) overnight at 4℃. After washing, membranes were incubated with HRP-conjugated secondary antibodies and detected using the ESCCL chemiluminescence reagent (Thermo Fisher). Band intensities were quantified with ImageJ software.

**RNA extraction and quantification**

RNA was extracted using the RNeasy Plus Mini Kit (Qiagen) and reverse transcribed to cDNA with the PrimeScript RT Reagent Kit (Takara). qPCR was performed using SYBR Green Master Mix (Takara) on an Applied Biosystems 7500 Real-Time PCR System. The following genes were analyzed: DUSP5, c-Myc, c-Fos, FOSL1, BCL-2, BCL-XL, CCND1, and ELK1. Relative expression levels were computed using the 2^−ΔΔCt^ method, with GAPDH as a reference.

**Immunofluorescence staining**

Immunofluorescence staining was implemented on fixed cells grown on coverslips. After blockade with 5% BSA, cells were added with primary antibodies against ERK1/2, VIM1, CDH1, ELK1, and DUSP5 overnight at 4℃. The following day, cells were probed with Alexa Fluor-conjugated secondary antibodies (Invitrogen) and stained with DAPI (Sigma). Images were captured using a confocal microscope (Leica TCS SP8). Fluorescence intensity was quantitated using ImageJ software.

**Chromatin immunoprecipitation (ChIP)-qPCR assays**

ChIP was performed with the ChIP-IT™ Express Kit (Active Motif). Briefly, cells were cross-linked with 1% formaldehyde and lysed to obtain chromatin, which was sonicated to achieve fragments of 200-500 bp. The chromatin was then immunoprecipitated using anti-ELK1 antibody (Cell Signaling Technology, #9182) or IgG. The enriched DNA was purified and analyzed by qPCR using primers targeting the DUSP5 promoter region. Binding sites were predicted on JASPAR (http://jaspar.genereg.net).

**Luciferase reporter assays**

The DUSP5 promoter region containing the ELK1 binding site was cloned into the pGL3 vector (Promega). HEK293T cells were co-transfected with the pGL3-DUSP5 promoter construct and a plasmid encoding ELK1 (GeneChem) using Lipofectamine 2000. After 48 h, cells were lysed, and luciferase activity was measured and normalized to Renilla luciferase activity.

**IHC** **for markers in mouse tumor tissues**

Tumor tissues were embedded in paraffin, sectioned (4 μm), and subjected to antigen retrieval by microwave heating in citrate buffer. Next, sections were blocked with 5% BSA and added with primary antibodies (KI67, C-Cas-3, VIM1, SNAI1, ERK1/2, and ELK1) overnight at 4℃. After washing, sections were probed with HRP-conjugated secondary antibodies, and the signal was visualized using DAB substrate (Vector Labs). Sections were counterstained with hematoxylin and imaged under a light microscope.

**Supplementary Figure 1.** Additional analysis of myeloid cell populations. A. UMAP plot showing trajectory analysis encompassing all inferred lineages. B. Scatter plots illustrating the expression of various cell-type markers along the two main differentiation trajectories. C. Bar plots comparing module scores of M1, M2, monocyte, and neutrophil markers across different samples. D-E. Cell communication network and bar plot depicting interactions within the EGF signaling pathway, highlighting the crosstalk between tumor/epithelial cells and immune cells, especially C2 and C3 macrophage subsets.

**Supplementary Figure 2.** Prognostic analysis of differentially expressed genes in C2 macrophages. A. Forest plot from univariate Cox regression identifying 21 prognostic genes. B-C. LASSO-Cox modeling results for prognostic gene selection and coefficient estimation. C. Lollipop plot showing coefficients of the 13 genes included in the final prognostic model. D. Forest plot of univariate Cox regression for the 13 model genes. E. Scatter plot of risk scores and survival times for TCGA ESCC patients. F. Heatmap of expression levels for the 13 prognostic genes across patients. G. Kaplan-Meier survival curves comparing high- and low-risk patient groups. H. Time-dependent ROC curves assessing model accuracy at 1, 3, and 5 years. I. Heatmap of gene set enrichment scores from MsigDB pathways significantly different between high- and low-risk groups. J-K. t-SNE plots and violin plots showing differences in six selected KEGG and GO Biological Process pathways between risk groups.

**Supplementary Figure 3.** Analysis of tumor/epithelial cell populations. A. UMAP plots showing tumor/epithelial cell clusters along with UMI counts, gene numbers, and cell cycle scores (G2M and S phase). B-D. UMAP plots dividing epithelial cells into two groups based on DUSP5 expression, boxplots comparing cell type proportions between groups, and bar plots showing cell type distribution across individual samples. E. Bubble plot showing marker gene expression for each tumor/epithelial cell subtype. F-I. Bubble and bar plots of five ERK/MAPK-related oncogenic pathway scores across epithelial subtypes and DUSP5 expression groups. J. Volcano plot displaying differentially expressed genes across epithelial cell clusters. K. Bar plot of GO enrichment analysis for dominant genes within each tumor/epithelial cell subtype.

**Supplementary Figure 4.** Macrophages activate ERK1/2 in ESCC cells through AREG. A-B. After treating TE-1 or KYSE30 cells with AREG for 0, 1.5, and 3 h, WB analysis was performed to detect ERK1/2 activation in USP5-OE or Empty vector cells. C-D. THP-1 cells were treated with PMA, IL-4, and M-CSF to induce the formation of M2 macrophages. These cells were co-cultured with DUSP5-OE or Empty vector TE-1 or KYSE30 cells, and WB was used to assess ERK1/2 activation in the TE-1 or KYSE30 cells. All cell-based experiments were performed with six independent biological replicates, and three technical replicates were conducted for each biological replicate. Statistical significance was evaluated using or two-way ANOVA with Tukey’s post-hoc test (B and D). P < 0.05 was considered statistically significant.
